# Exogenous γ-Aminobutyric Acid (GABA) Application Mitigates Salinity Stress in Maize Plants

**DOI:** 10.3390/life12111860

**Published:** 2022-11-12

**Authors:** Bandar S. Aljuaid, Hatem Ashour

**Affiliations:** 1 Department of Biotechnology, College of Science, Taif University, P.O. Box 11099, Taif 21944, Saudi Arabia; 2 Department of Agricultural Botany, Faculty of Agriculture, Ain Shams University, Cairo 11566, Egypt

**Keywords:** abiotic stress, antioxidants enzymes, gene expression, NaCl

## Abstract

The effect of γ-Aminobutyrate (GABA) on maize seedlings under saline stress conditions has not been well tested in previous literature. Maize seedlings were subjected to two saline water concentrations (50 and 100 mM NaCl), with distilled water as the control. Maize seedlings under saline and control conditions were sprayed with GABA at two concentrations (0.5 and 1 mM). Our results indicated that GABA application (1 mM) significantly enhanced plant growth parameters (fresh shoots and fresh roots by 80.43% and 47.13%, respectively) and leaf pigments (chlorophyll a, b, and total chlorophyll by 22.88%, 56.80%, and 36.21%, respectively) compared to untreated seedlings under the highest saline level. Additionally, under 100 mM NaCl, methylglyoxal (MG), malondialdehyde (MDA), and hydrogen peroxidase (H_2_O_2_) were reduced by 1 mM GABA application by 43.66%, 33.40%, and 35.98%, respectively. Moreover, maize seedlings that were treated with 1 mM GABA contained a lower Na content (22.04%) and a higher K content (60.06%), compared to the control under 100 mM NaCl. Peroxidase, catalase, ascorbate peroxidase, and superoxide dismutase activities were improved (24.62%, 15.98%, 62.13%, and 70.07%, respectively) by the highest GABA rate, under the highest stress level. Seedlings treated with GABA under saline conditions showed higher levels of expression of the potassium transporter protein (*ZmHKT1*) gene, and lower expression of the *ZmSOS1* and *ZmNHX1* genes, compared to untreated seedlings. In conclusion, GABA application as a foliar treatment could be a promising strategy to mitigate salinity stress in maize plants.

## 1. Introduction

Due to the increasing population of the world, and the demands for food, salinity is regarded as one of the most serious global issues confronting agricultural sectors in recent decades [1]. Salinity affects over 1125 million hectares worldwide, and that number is predicted to increase annually as a result of human activity [2]. It has been well known that plants exposed to abiotic stress, such as saline and drought conditions, produce an abundance of reactive oxygen species (ROS), including superoxide anion, hydrogen peroxide, and hydroxyl radical [3,4]. Consequently, the overproduction of reactive oxygen species causes lipid peroxidation, metabolic disturbances, and even cell death [5]. Plants suffer from major problems when grown under saline conditions, including water deficiency caused by osmotic stress, disruption of the plant’s ionic systems, and ion toxicity [6,7]. High Na^+^ and Cl ion concentrations in soil or water reduce net photosynthesis [8] and the concentration of several compounds, such as sugars, polyamines, and amino acids [9].

Plant salt tolerance is a multifaceted trait that involves numerous biochemical and physiological mechanisms. It is critical for breeding improved varieties to identify multiple genes whose expression allows plants to adapt to, or tolerate, different levels of salt [10]. There are some methods to control salinity, including installing new irrigation and drainage systems [11], breeding plants [12], root treatments by some types of fungi [13], and applying biostimulants to improve the plant’s defense system, such as salicylic acid [14].

One of the most important cereal crops grown around the world is maize (*Zea mays*), which is classified as a moderately sensitive crop to salinity [11]. Because of its high starch content, it is quickly becoming one of the main ingredients in foods, textiles, biofuels, silage, and many other industries. [15]. It has been well known that maize suffers from severe damage and significant losses in growth and productivity when grown in saline conditions [16]. More than 250 mM NaCl of salinity inhibits maize plant growth, causing severe wilting [17]. As a result, the aim of the current research was to find a method that could mitigate or minimize the negative impact of salts on maize plants.

Gamma-aminobutyric acid (GABA) is widely distributed in plants and is associated with stress responses, signaling, and storage [18]. Previous works indicated that GABA is involved in many plant processes and reactions, such as the oxidative stress defense [19], the balance of the C/N ratio [20], signaling function [21], and the control of osmotic pressure [22]. Under abiotic stresses such as salinity and drought, GABA is accumulated in the plant [23,24]. Previous reports indicated that the use of a GABA improved salinity stress tolerance by improving antioxidant enzyme activity and reducing reactive oxygen species accumulation in plants [25], maintaining the C/N ratio in balance, regulating cell osmosis [26], controlling Na^+^ and K^+^ absorption [27], and increasing some bioactive compounds such as phenolic compounds, proline, and amino acids [28]. In a previous work [29], treating seedling maize roots (root drenching) with a GABA application under saline conditions (150–300 Mm NaCl) led to an increase in seedling growth, proline and sugar content in shoots, and enzymatic antioxidant activity compared to untreated plants. To the best of our knowledge, the influence of GABA treatment via a foliar application on the growth, physiological, and molecular changes of maize plants has not been studied before. Thus, the current study was performed to investigate the morphological, physiological, and molecular responses of maize plants treated with GABA as a foliar application under saline conditions.

## 2. Materials and Methods

### 2.1. Seed Preparation and Materials

Maize grains hybrid (Hytech 2030) obtained from Misr Hytech Seed Int. (Cairo, Egypt) were soaked in NaOCl (0.5%) for 5 min for sterilization. The gains were washed 3 times with distilled water and left to dry for 2 h before germination.

### 2.2. Seed Germination

On wet filter paper, maize grains were allowed to germinate for 24 h at 25 °C. Five seedlings were placed in each of 8 Kg of pre-washed sand-filled black plastic pots (20 cm in diameter) after being chosen for their uniform size. After planting, the pots were kept in a growth chamber at 27/17 °C day/night with 170 µmol m^−2^ S^−1^ light intensity and 70–75% RH. The pots were irrigated every two days with a half-strength Hoagland’s solution.

### 2.3. Salt Treatments

After 14 days, the pots were split into two main groups: saline and control conditions. A half-strength Hoagland’s nutrient solution was used to irrigate seedlings from the saline group that contained NaCl salt with desired concentrations (50 and 100 mM NaCl), while those of the unstressed group (control, 0 mM NaCl) were irrigated with Hoagland’s nutrient solution (half strength).

### 2.4. GABA Foliar Application

To apply the GABA treatment, the pots (from every salinity level) were divided into three subgroups, and the following solutions were used: 0.5 mM GABA, 1 mM GABA, and control plants (sprayed with distilled water). The spraying solution was 25 mL/pot and was repeated five times at 15, 17, 19, 21, and 24 days after transplanting with Tween-20 (0.05%). The pots were arranged in a completely randomized design (CRD) with three replicates. After 7 days from the last GABA application (31 days), the seedlings were harvested for morphological, chemical, and molecular determination.

### 2.5. Seedlings Growth Parameters and Pigments

A digital balance was used to determine the fresh shoot and root. The methods described by Lichtenthaler and Wellburn [30] were followed to determine chlorophyll a, chlorophyll b, total chlorophyll, and carotenoids.

### 2.6. Determination of Cell Membrane Integrity

To determine the cell membrane stability index, the method of Abd Elbar et al. [31] was used. In brief, eight leaf discs were incubated in 10 mL of deionized water and kept for 24 h on a shaker. After that, *EC* meters were used to determine *EC*_1_ values, and then samples were autoclaved for 20 min at 120 °C to determine the values of *EC*_2_. The following equation was used to calculate the cell membrane stability index:

(1) $$\mathrm{MSI}=[1\;–\;(\frac{\mathrm{EC}1}{\mathrm{EC}2})]\;\times \;100$$

whereas MSI: Membrane stability index; EC1: Electrical conductivity after incubated and before autoclaved; EC2: Electrical conductivity after autoclaved.

### 2.7. Determination of Methylglyoxal Content, Hydrogen Peroxide, and Malondialdehyde

According to Hossain et al. [32], the methylglyoxal content (MG) was determined using a UV-spectrophotometer at 335 nm. Hydrogen peroxide (H_2_O_2_) content was determined calorimetrically by the K iodide method [33], as described previously by Nasser et al. [34]. The thiobarbituric acid method (TBA) was used to determine malondialdehyde (MDA), according to the method of [35].

### 2.8. Determination of Antioxidant Enzymes Activates

Ascorbate peroxidase (APX) activity was assessed as described previously by Doklega et al. [36], with some modifications. The decrease of absorbance at 290 nm was monitored for 3 min. The reaction mixture with a total volume of 3 mL included 100 µL crude enzyme, 50 mM phosphate buffer (pH 7), 0.1 mM EDTA, 0.5 mM ascorbic acid, and 0.1 mM H_2_O_2_. The addition of H_2_O_2_ initiated the reaction. One enzyme activity unit was defined as the amount of enzyme required for the oxidation of 1 µmol of ascorbate per minute. The rate of ascorbate oxidation was calculated using the extinction coefficient (ε = 2.8 mm^−1^ cm^−1^). Catalase (CAT) activity was measured according to a previous report [37]. The reaction mixture with a total volume of 3 mL contained 15 mM H_2_O_2_ in 50 mM phosphate buffer (pH = 7). The reaction was initiated by adding 50 μL crude enzyme. The activity was calculated from the extinction coefficient (ε = 40 mM^−1^ cm^−1^) for H_2_O_2_. One unit of enzyme activity was defined as the decomposition of 1 μmol of H_2_O_2_ per minute at 240 nm. Guaiacol peroxidase (G-POX) activity was assessed by tracking the rise in absorbance (470 nm) as guaiacol was transformed into tetraguaiacol [38]. The assay mixture (100 mL) contained 10 mL of 1% (*v*/*v*) guaiacol, 10 mL of 0.3% H_2_O_2_, and 80 mL of 50 mM phosphate buffer (pH = 6.6). The volume of 100 µL of the crude enzyme was added to 2.9 mL of the assay mixture to start the reaction. The absorbance was recorded every 30 s for 3 min at 470 nm. To determine superoxide dismutase (SOD), the method of Beyer and Fridovich [39] was followed. The reaction mixture with a total volume of 3 mL contained 100 μL crude enzyme, 50 mM phosphate buffer (pH 7.8), 75 μM NBT, 13 mM L-methionine, 0.1 mM EDTA, and 0.5 mM riboflavin. The addition of riboflavin initiated the reaction. Then, the reaction mixture was illuminated for 20 min with a 20 W fluorescent lamp. One enzyme activity unit was defined as the amount of enzyme required to result in a 50% inhibition in the rate of nitro blue tetrazolium (NBT) reduction at 560 nm.

### 2.9. Determination of Na and K

The flame photometric (Jenway, Leicestershire, UK) method described by Havre [40] was used to determine the Na and K mineral concentrations in the leaves.

### 2.10. Gene Expression

An RNA extraction kit (Sigma–Aldrich, St. Louis, MO, USA) was used to extract the total mRNA from different treatments according to the manufacturer’s protocol. To determine the RNA purification after the reverse transcription of RNA and cDNA formation, a NanoDrop™2000/2000c spectrophotometer was used according to the manufacturer’s protocol (Promega, Walldorf, Germany). Real-time PCR (Rotor-Gene 6000, Hilden, Germany) was used to run realtime quantitative reverse-transcription polymerase chain reaction (qRT-PCR) on diluted cDNA in triplicate, and the primer sequences used in qRT-PCR are provided in Table 1. The reference gene (β-Actin housekeeping gene) was utilized to analyse gene expression using SYBR^®^ Green. The relative gene expression was determined using the 2∆DDCt method [41].

### 2.11. Statistical Analysis

All data were subjected to one way ANOVA. Tukey’s multiple range test (*p* ≤ 0.05) was used by using SAS software to analyse differences in means and ± SE from six replicates.

## 3. Results

### 3.1. Effect of GABA on Growth and Pigments under Salinity Condition

**Figure 1:**
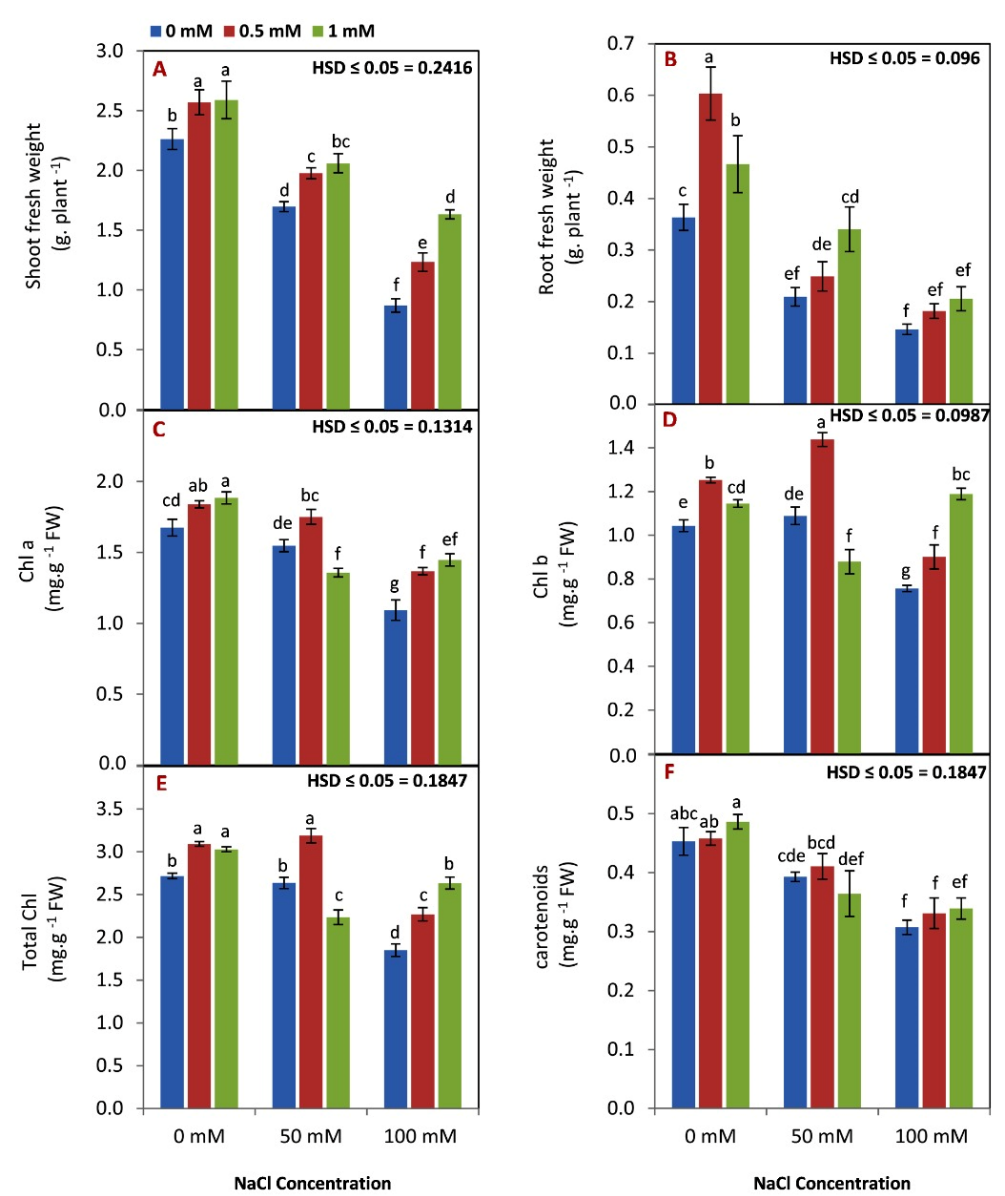
Effect of GABA (0, 0.5, 1 mM) and salt stress (0, 50, 100 mM) interaction on shoot fresh weight (**A**), root fresh weight (**B**), chlorophyll a content (**C**), chlorophyll b (**D**) content, total chlorophyll (**E**), and carotenoid content (**F**) of leaves of maize seedlings under three level of salinity (0, 50, 100 mM). Different letters between all values are significantly different at *p* < 0.05 (n = 6 ± SE) according to the Tukey test.

In Figure 1A,B there is a clear trend of decreased growth of maize seedlings that were exposed to saline stress compared to the control plants. Another important finding was that both rates of GABA application reduced the harmful effect of salinity stress on the growth of maize seedlings (fresh and dry weights) under either the saline condition or the control condition.

From the data in Figure 1C,D, it is apparent that chlorophyll a, chlorophyll b, and total chlorophyll contents were decreased by increasing the salinity level. Further, GABA application at the highest rate (1 mM) showed a positive effect on the content of all chlorophyll parameters compared to the low GABA rate (0.5 mM) and untreated plants.

The results in Figure 1E indicated that carotenoid content in maize seedlings decreased with increasing salinity levels. The results did not detect any significant difference in carotenoid content by GABA application under normal or saline conditions.

### 3.2. Effect of GABA on the Membrane Stability Index (CMSI), Methylglyoxal Content (MG), H_2_O_2_, and Malondialdehyde Content (MDA) under Saline Condition

**Figure 2:**
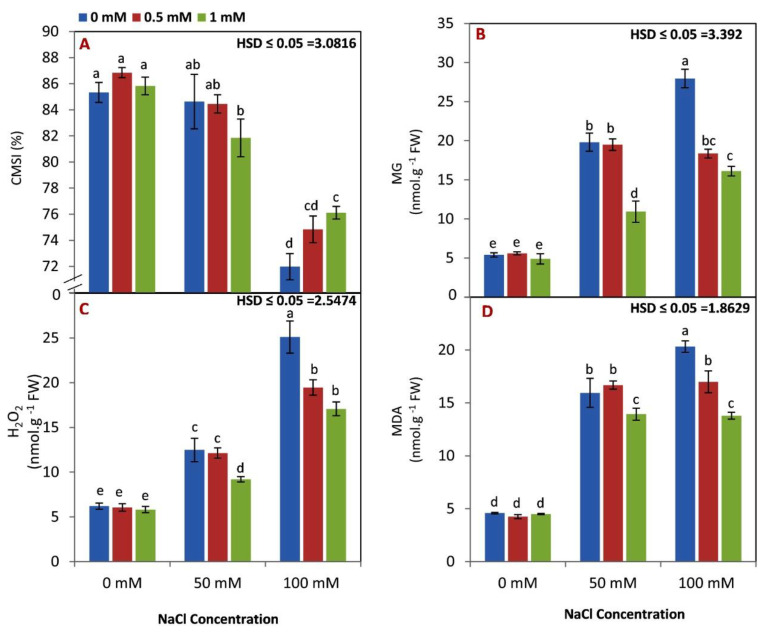
Effect of GABA (0, 0.5, 1 mM) and salt stress (0, 50, 100 mM) interaction on CMSI (%) (**A**), MG (**B**), H_2_O_2_ (**C**), and MDA (**D**) content in the leaves of maize seedlings under two salinity levels. Different letters between all values are significantly different at *p* < 0.05 (n = 6 ± SE) according to the Tukey test.

A significant decrease in CMSI was observed with increasing salinity levels (Figure 2A). There was no significant difference in CMSI for all rates of GABA application under 0 and 50 mM NaCl levels. However, both doses of GABA (0.5 and 1 mM) conserved CMSI compared to nontreated seedlings under a 100 mM NaCl level.

As expected, MG, H_2_O_2_, and MDA were increased by increasing the level of salinity from 0 to 100 mM NaCl (Figure 2B–D). There were no differences between 0.5 and 1 mM of GABA application on MG, H_2_O_2_, and MDA under nonsaline conditions. However, the high rate of GABA was effective for reducing MG, H_2_O_2_, and MDA levels under 50 mM NaCl. Additionally, MG, H_2_O_2_, and MDA levels of GABA treated seedlings were lower than those of seedlings of control plants under 100 mM NaCl.

### 3.3. Effect of the GABA Application on the Activities of Antioxidant Enzymes

**Figure 3:**
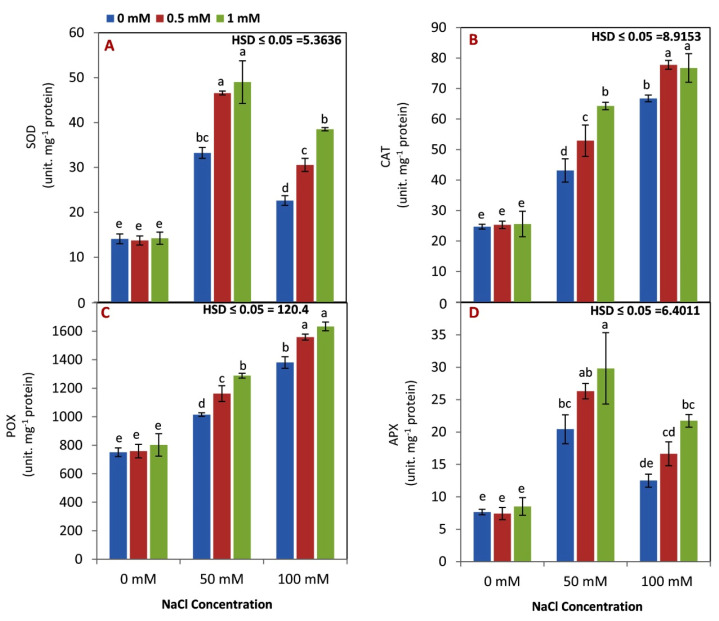
Effect of GABA application (0, 0.5, 1 mM) and salt stress (0, 50, 100 mM) interaction on the activity of SOD (**A**), CAT (**B**), POX (**C**), and APX (**D**) activities in the leaves of maize seedlings under two salinity levels. Different letters between all values are significantly different at *p* < 0.05 (n = 6 ± SE) according to the Tukey test.

Under the nonsaline condition, no differences were observed in the activities of SOD, CAT, POX, and APX (Figure 3A–D). However, the activity of CAT and POX enzymes was increased by increasing the salinity level from 50 to 100 mM NaCl, while SOD and APX were decreased. The antioxidant activities of all enzymes were increased by increasing the rate of GBAB application from 0 to 1 mM.

### 3.4. Effect of GABA Application on Na, K, and Na/K Ratio

**Figure 4:**
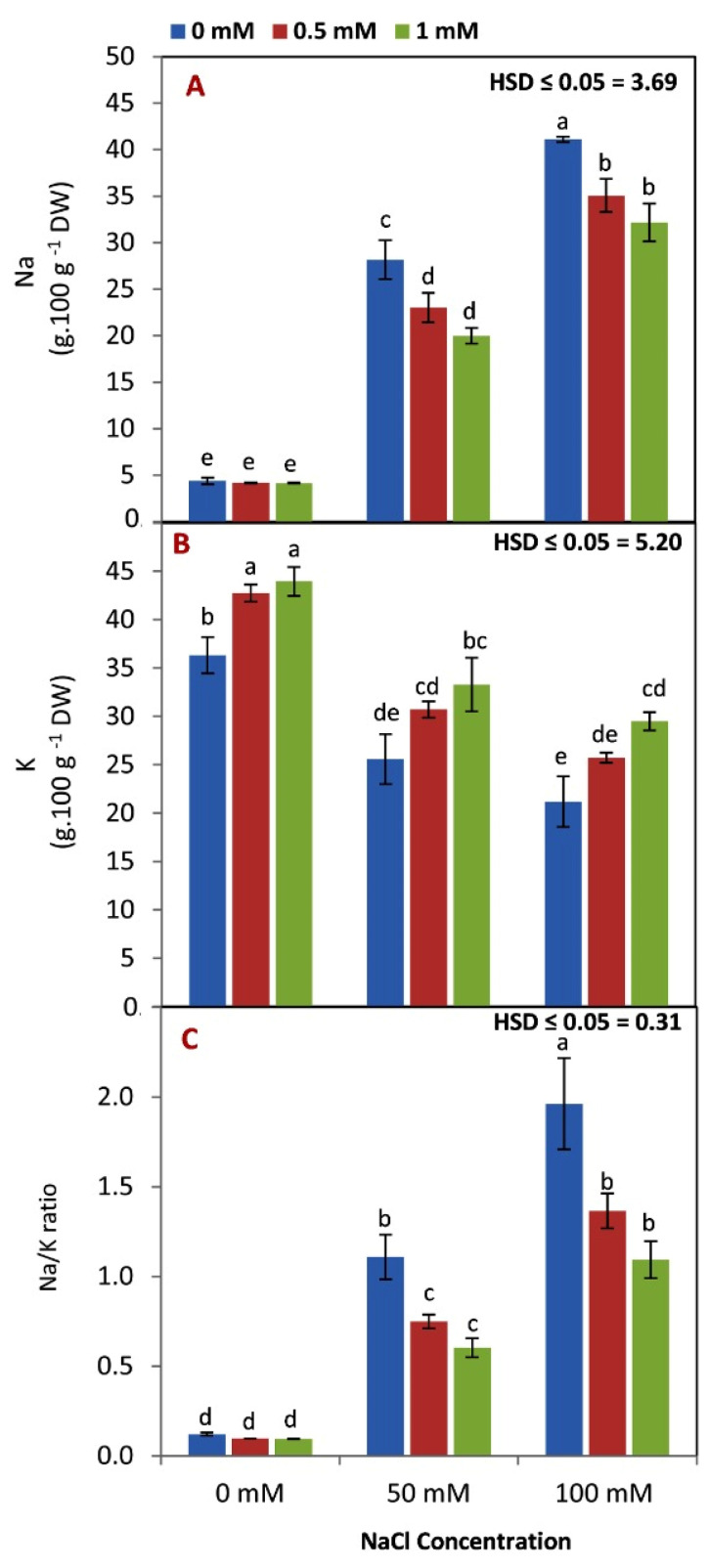
Effect of GABA application (0, 0.5, 1 mM) and salt stress (0, 50, 100 mM) interaction on Na^+^ (**A**), K^+^ (**B**), and Na/K ratio (**C**) content in the leaves of maize seedlings under two salinity levels. Different letters between all values are significantly different at *p* < 0.05 (n = 6 ± SE) according to the Tukey test.

As expected, Na content and the Na/K ratio increased while the K content decreased with increasing salinity levels (Figure 4A–C). The most interesting result was that N content, and the Na/K ratio, were decreased (under salinity stress conditions) in seedling leaves by increasing the rate of GABA application from 0 to 1 mM. Additionally, K content was increased by increasing GABA levels under controlled or saline conditions. Under nonsaline conditions, GABA application didn’t affect the Na content and Na/K ratio in seedling leaves.

### 3.5. Effect of GABA Application on the Genes Expression

**Figure 5:**
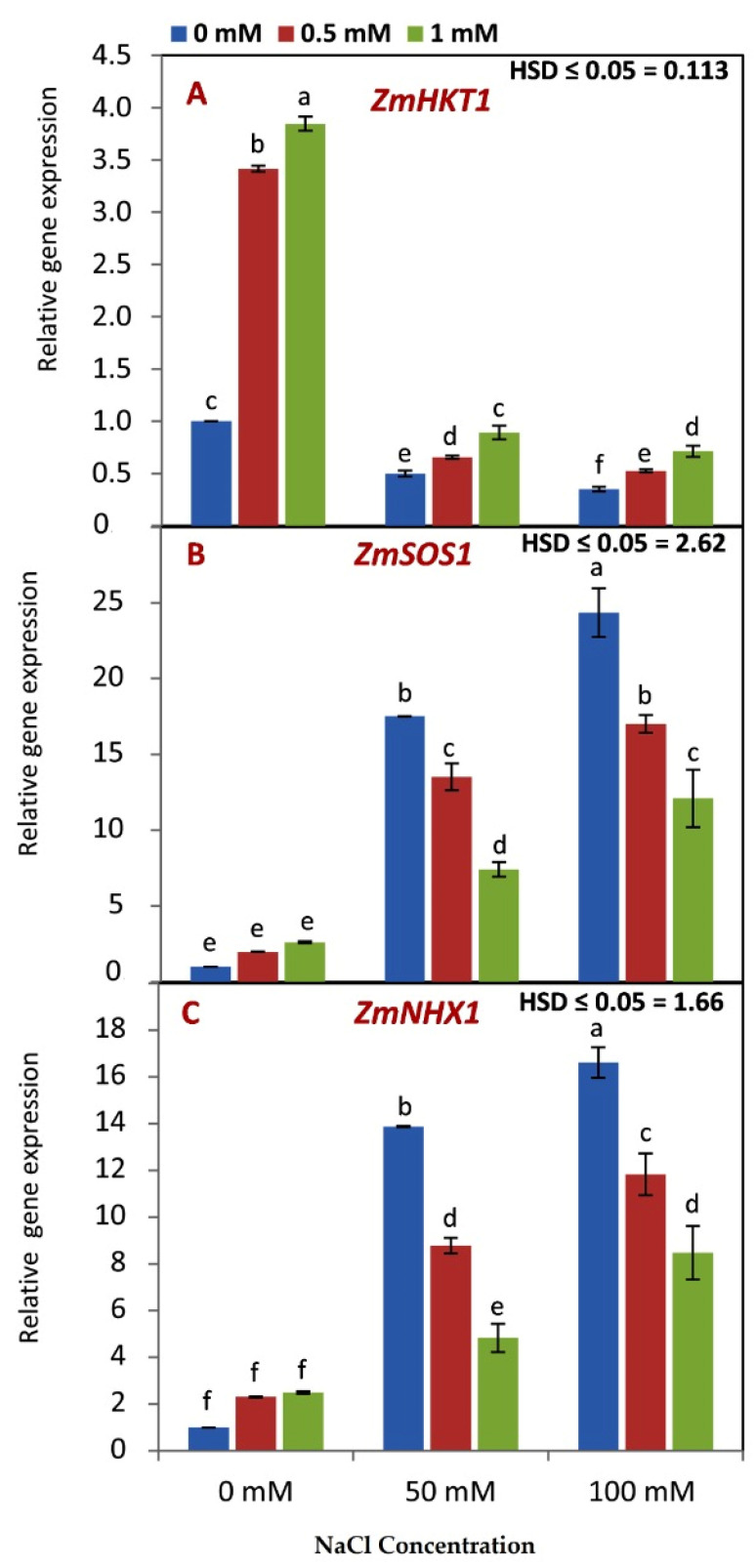
Effect of GABA application (0, 0.5, 1 mM) and salt stress (0, 50, 100 mM) interaction on gene expression of *ZmHKT1* (**A**), *ZmSOS1* (**B**), and *ZmNHX1* (**C**) in leaves of maize seedlings under two salinity levels. Different letters between all values are significantly different at *p* < 0.05 (n = 6 ± SE) according to the Tukey test.

As shown in Figure 5A, the expression of the *ZmHKT1* gene was higher in seedlings that were treated with both GABA concentrations compared to the nontreated seedlings under normal and nonsaline conditions. The expression of the *ZmHKT1* gene was decreased by increasing salinity levels.

The *ZmSOS1* and *ZmNHX1* genes showed a similar profile when seedlings treated with both GABA concentrations were compared to nontreated seedlings under nonsaline conditions (Figure 5B,C). The expression of both genes was increased by increasing salinity levels. Under 50 and 100 mM NaCl, increasing GABA rates resulted in the reduced expression of both the *ZmSOS1* and *ZmNHX1* genes.

## 4. Discussion

Plants exposed to salt stress, in this study, showed a decline in plant growth (shoot and root fresh weights, Figure 1A,B). These results seem to be consistent with previous research, which found that salinity reduces plant growth by several mechanisms, including osmotic stress [1], ionic toxicity [6], reduced photosynthesis [8], reduced cell division, and nutrient uptake [42,43]. Under normal and salt stress conditions, it was detected that plants treated with GABA showed a significant improvement in plant growth. The findings of the current study are consistent with those of Jin et al. [18], who found that exogenous GABA application enhanced the growth (fresh weight, dry weight, and leaf area) of watermelon seedlings under saline stress conditions. A possible explanation for these results may be due to the fact that GABA plays a role in reducing the reduction in net photosynthesis under saline conditions [44]. Additionally, previous work indicated that endogenous GABA could enhance the plant’s tolerance to stresses [45]. Accordingly, the exogenous GABA could, by foliar treatment, increase the level of GABA inside the plant tissue, resulting in higher tolerance to salinity stress [18]. The results of this study show that chlorophyll a, b, total chlorophyll, and carotenoids (Figure 1C–F) were significantly decreased by saline stress. This finding is in agreement with the Nasrallah et al. [46] findings, which showed that total chlorophyll and carotenoids in board bean plants were decreased by increasing the salinity level. This result might be due to the fact that salinity enhances membrane breakdown and the activation of chlorophyllase enzymes leads to an inhibition of chlorophyll synthesis [47].

Under abiotic stress conditions, some biochemical markers (such as ROS, OH, O_2_^−^, and H_2_O_2_) were triggered in plants and worked as signaling molecules to enhance the plant defense system [48]. However, the massive accumulation of these markers could negatively affect some physiological and chemical processes in plants [49]. GABA may have been an important factor in elevating the activity of antioxidant enzymes [50]. Additionally, it has been found that exogenous GABA applications protect plants from oxidative damage in some crops, such as peaches [44,51]. By influencing some physiological processes such as photosynthesis, stomatal movement, and root differentiation, methylglyoxal (MG) has been proven to be harmful at high concentrations [52]. Our results in Figure 2B show that MG was increased under saline conditions. Previous works also observed an increase in MG content in wheat and rice plants under NaCl stress [53,54]. In our study, the GABA application reduced MG content in maize seedlings. This result could be due to the role of GABA in reducing ROS. In this study, the GABA application reduced H_2_O_2_ content in seedlings irrigated with saline water (Figure 2C). Similar findings have been observed by [18]. Our results in Figure 2D indicated that MDA content was reduced by GABA application under saline conditions. This result is supported by previous studies [55,56].

Our results in Figure 3A–D show that antioxidant enzyme activity was increased by salinity stress. Many previous reports emphasize that antioxidant enzymes in plants are increased under stressful conditions to help plants resist unfavorable conditions [57,58]. GABA has been shown to play an important role in the regulation of reactive oxygen species (ROS) scavengers in plant systems [59]. It has been known that SOD is responsible for converting O_2_^−^ to H_2_O_2_, while converting H_2_O_2_ to H_2_O and O_2_ is controlled by CAT, APX, and POX enzymes [60]. In this study, and a previous study [18], GABA treatment enhanced the activity of SOD under saline conditions. Additionally, GABA as a foliar application is also linked with controlling SOD and APX activity in some crops, such as tomatoes [31]. Redox balance in leaves is critical for maintaining the maximum efficiency of metabolic enzymes [18,61].

In many crops, such as barley and white cover, the ratio of Na/K can be utilized as a physiological index for salt sensitivity [27,62]. Additionally, the cytosolic Na/K ratio is maintained by the concentration of Na, which interacts with K, and is vital for the salinity tolerance mechanism given its involvement in multiple metabolic activities [63]. In this study, the data in Figure 3A–C indicate that GABA application reduced the Na level and Na/K ratio under saline stress. GABA application reduced the accumulation of N in roots under saline stress. The ability of GABA to reduce N content and Na/K ratio under saline conditions could be attributed to its role in mitigating oxidative damage by osmotic regulation [27]. Additionally, GABA could prevent lipid peroxidation and sustain hormone and mineral nutrition under a variety of environmental conditions [64]. GABA also has an impact on several physiological processes, including cell division, metabolic regulation, and detoxifying techniques, which enable plants to endure and grow in stressful environments, including salinity [61].

The expression of the *ZmSOS1* and *ZmNHX1* genes, as well as the opposite trend of the *ZmHKT1* gene, can help plants survive under saline stress by excluding Na from the cytosol to the apoplast or vacuole, protecting cytosolic enzymes from Na ion toxicity. Conversely, GABA application led to over expression of *ZmHKT1*, and down expression of *ZmSOS1* and *ZmNHX1.* A previous study found that the expression of the *SOS1* salt gene was increased by salt stress [65,66]. These results suggested that GABA can decrease the Na/K ratio selectivity and photosynthetic capacity of maize plants under saline stress conditions. Our results in Figure 1 and Figure 4 confirmed our hypothesis that GABA application enhanced the photosynthesis process and reduced the Na/K ratio.

## 5. Conclusions

**Figure 6:**
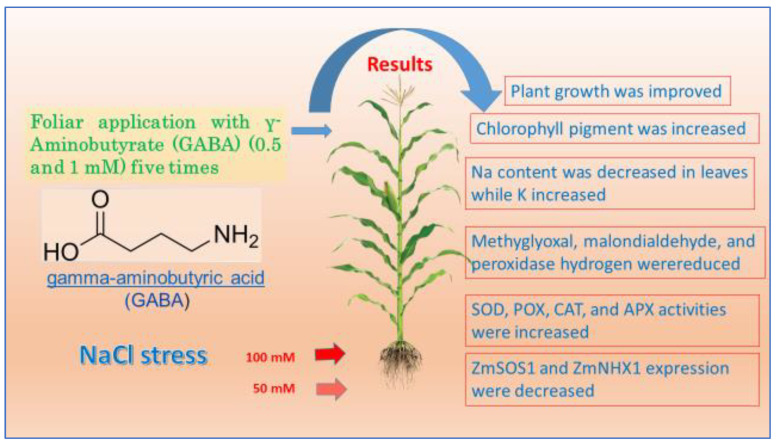
Summarized chart of the effect of GABA application on the morphological, physiological, and molecular traits of maize seedlings under saline stress.

The current study shows that GABA as a foliar application could be useful to enhance the growth of maize seedlings and mitigate saline stress (Figure 6). GABA application is intended to regulate and control some measured parameters, including plant growth, chlorophyll pigments, the activity of antioxidant enzymes, and related gene expression under saline stress. Additionally, Na content in maize seedlings was decreased while K absorption was increased by GABA application under salinity conditions. This demonstrates that GABA reduces Na accumulation and isolates it in vacuoles in the leaves of maize seedlings under saline stress. Further investigations are required to investigate the effect of GABA on the production and quality of maize plants. The current work may also have agronomic significance for the use of GABA as a foliar application in the management of salt-sensitive crops in salinity-affected soils in arid and semiarid regions.

## Figures and Tables

**Table 1:** List of primers.

Primer Name		Sequence	Tm
ZmHKT1	F	5′-TGCTAATGTTTATCGTGCTG-3′	56 °C
	R	5′-AGGCTGATCCTCTTCCTAAC-3′	
ZmSOS1	F	5′-ACTTGCAGGAGGAATACAAC-3′	
	R	5′-CGAGAAGAGAAGACCACATC-3′	
ZmNHX	F	5′-CGTGATGTCGCATTACACCT-3′	
	R	5′-CTGGCAAACTCCCACTTCTC-3′	
β-Actin	F	5′-GTGCCCATTTACGAAGGATA-3′	
	R	5′-GAAGACTCCATGCCGATCAT-3′	

## Data Availability

Not applicable.
